# Promoting smoking cessation and preventing relapse to tobacco use following a smokefree mental health in‐patient stay: A multi‐centre randomised controlled feasibility study

**DOI:** 10.1111/add.70519

**Published:** 2026-08-02

**Authors:** Petal Petersen Williams, Lisa Huddlestone, Emily Shoesmith, Jennifer Sweetman, Heidi Stevens, Alex Mitchell, Fraser Wiggins, Lesley Sinclair, Victoria Exley, Catherine Hewitt, Judith Watson, Qi Wu, Jodi Pervin, Moira Leahy, Claire Paul, Lion Shahab, Michelle Horspool, Tim Coleman, Simon Hough, Elena Ratschen, the SCEPTRE Collaboration team, Lesley Colley, Samantha Brady, John Britton, Simon Gilbody, Steve Parrott, Peter Coventry, Gregor Russell, Paul Galdas

**Affiliations:** ^1^ Mental Health and Addiction Research Group, Department of Health Sciences University of York York UK; ^2^ Mental Health, Alcohol, Substance Use and Tobacco Research Unit South African Medical Research Council South Africa; ^3^ Institute for Life Course Health Research, Department of Global Health Stellenbosch University Stellenbosch South Africa; ^4^ York Trials Unit, Department of Health Sciences University of York York UK; ^5^ Research Development Unit Sheffield Health Partnership University NHS Foundation Trust Sheffield UK; ^6^ Research and Development Leeds and York Partnership NHS Foundation Trust Leeds UK; ^7^ Institute for Epidemiology and Health Care University College London London UK; ^8^ Centre for Academic Primary Care University of Nottingham Nottingham UK; ^9^ Independent PPI Co‐applicant Cheshire UK; ^10^ Research and Development Tees, Esk and Wear Valleys NHS Foundation Trust Darlington UK; ^11^ University of Nottingham UK; ^12^ School of Nursing and Public Health Manchester Metropolitan University Manchester UK; ^13^ Research and Knowledge Service Bradford District Care NHS Foundation Trust Bradford UK

**Keywords:** feasibility, in‐patient, mental health, smoking, smoking cessation, tobacco dependence

## Abstract

**Aims:**

This study assessed the feasibility and acceptability of a new theory‐ and evidence‐based intervention designed to prevent return to pre‐admission smoking behaviours after discharge from a smokefree mental health in‐patient setting in the United Kingdom (UK).

**Design:**

A multi‐centre individually randomised controlled feasibility trial with follow‐up at 3 and 4–6 months.

**Setting:**

Acute adult mental health wards of six National Health Service Mental Health Trusts in England.

**Participants:**

Thirty‐eight (17 intervention, 21 usual care) adults who smoked on or after admission to an acute adult mental health in‐patient ward and wished to continue reducing or quitting smoking after discharge, enrolled between February and November 2024.

**Intervention:**

The intervention comprised usual care and a 12‐week, theory‐ and evidence‐informed support programme including a bespoke resource kit, personalised behavioural support including phone calls and text messages, and access to a digital smoking cessation app with 24/7 live support. It was delivered by trained mental health workers. The comparator group received usual care as per UK national guidance, typically involving brief behavioural support, offers of nicotine replacement therapy and sometimes electronic cigarettes.

**Measurement:**

Primary outcomes included recruitment and retention, intervention acceptability and feasibility of collecting smoking, mental health and economic data. Success criteria for progression to trial were set a priori at a minimum of 60% of the *n* = 64 target for both recruitment and retention, i.e. at *n* = 38. Secondary outcomes included measures of smoking, quitting, reduction of cigarette consumption. Acceptability data were collected through exploratory interviews with participants, mental health staff and interventionists.

**Findings:**

We recruited *n *= 38 (60%) participants and retained *n* = 20 (52.6%) at 3 months, and *n* = 13 (34.2%) at second follow‐up. As such, criteria agreed to determine progression to full trial were marginally met for recruitment and not met for retention. Two intervention participants reported maintaining abstinence. Motivation to quit remained higher in the intervention than the usual care group over time, and use of e‐cigarettes was overall common (76.9% *n* = 10 across groups at 4–6 month follow up). Intervention group participants reported valuing the tailored and personalised behavioural support options but engaged little with tailored digital tools. Health economic data collection was feasible, though refinement is needed for future research.

**Conclusions:**

A new intervention delivering smoking cessation support to individuals discharged from smoke‐free mental health settings was discontinued because key feasibility criteria were not met, highlighting the need for focused reconsideration of recruitment and retention strategies, intervention design and delivery in mental health in‐patient populations. Design implications are discussed, taking into account individual patient‐level and systemic challenges.

## BACKGROUND

Tobacco smoking is a leading cause of preventable death and disease and was responsible for 7.7 million deaths globally in 2019 [1], with a disproportionate burden on individuals with mental health conditions [2, 3]. In the United Kingdom (UK), smoking rates among those with mental illness remain significantly higher than in the general population [4, 5], particularly among hospitalised patients, where prevalence can reach over 70% [5, 6]. Addressing tobacco dependence in this group is crucial for reducing health inequalities and improving long‐term outcomes.

Evidence indicates that smoking cessation benefits both physical and mental health, reducing depression, anxiety, and stress [7, 8]. However, individuals with mental illness face unique challenges to stop smoking, including higher nicotine dependence [9], social norms around smoking in mental health settings [5, 10, 11], and limited access to tailored support [12, 13, 14]. Smoking has historically been deeply ingrained in the culture of psychiatric care, where it was often perceived as a coping mechanism [10, 15], or a social activity [16]. The challenges associated with attempts to stop smoking in this population are compounded by disparities in healthcare access. Research suggests that individuals with severe mental illness receive fewer smoking cessation interventions than those without mental health conditions, despite having a greater need for support [17]. Additionally, mainstream cessation services may not be adequately tailored to address the unique barriers faced by people with mental illness, including concerns about the impact of smoking cessation on mental health and the need for integrated, personalised support [18]. Consequently, interventions must account for the complex interplay between smoking behaviours, mental health symptoms, potential interactions between tobacco smoke and some psychoactive medications, and broader social determinants of health.

While many hospitals worldwide, including mental health in‐patient settings, are now ‘smokefree’ and support patients to achieve abstinence when admitted, relapse commonly occurs within days of discharge [19, 20]. In the UK, national guidance mandates smokefree services and tobacco dependence treatment [21]; however, post‐discharge support remains limited.

The Promoting Smoking Cessation and Preventing Relapse to Tobacco use following a smokefree mental health in‐patient stay (SCEPTRE) research programme was designed to address this gap by providing structured, post‐discharge support to prevent relapse and promote sustained smoking cessation following a smokefree in‐patient stay [22, 23]. A detailed description of the intervention and its development is provided elsewhere [23].

This study aimed to evaluate acceptability of the intervention, feasibility of recruitment and retention, preliminary smoking outcomes, and the viability of collecting health economic data to inform a future trial.

## METHODS

This manuscript follows the Consolidated Standards of Reporting Trials (CONSORT) guidelines for pilot and feasibility trials [24] (see Data S1). The methods and outcome measures are summarised below and described in full in the published protocol [25].

### Trial design

A multi‐centre, parallel group, individually randomised controlled feasibility trial was conducted between February and November 2024 (February to July for recruitment, and July to November for follow‐up). Participants were randomised prior to discharge to receive either a 12‐week, theory‐and evidence‐informed behavioural support programme to promote smoking cessation and prevent relapse, or usual care. Follow‐up assessments were undertaken at 1) three months and 2) between four and six‐months post‐randomisation.

### Patient and public involvement and engagement (PPIE)

The SCEPTRE PPIE panel included three individuals with lived experience of mental health in‐patient care and tobacco dependence, and one carer. Members were involved from the outset, co‐producing participant materials, advising on recruitment, and contributing to the study website and inclusive language guidance to support recruitment. The panel met regularly, providing feedback and input through meetings, check‐ins, and informal discussions to support ongoing revisions and flexible participation. Further details of the PPIE process are reported elsewhere [26].

### Study setting

Participants were recruited from 25 acute adult mental health inpatient wards of six English NHS Mental Health Trusts located in Yorkshire, Leicestershire, Hertfordshire, Derbyshire, East London and Oxfordshire, with patient populations representing a wide range of demographic and clinical characteristics across diverse urban and rural catchment areas. To be eligible to participate, Trusts had to provide evidence that they were at a minimum working towards full implementation of smokefree policy in line with national guidance [21]. Due to national tobacco control policy developments, focused intensive support had been increasingly implemented in acute mental health settings while this feasibility study was in preparation. Trusts involved in the implementation and evaluation of newly funded intensive support programmes were excluded.

### Participants

Patients aged ≥18 years admitted to an acute adult mental health ward who smoked on or at any point after admission and expressed interest in maintaining abstinence/cessation, reducing smoking, or working towards these goals after discharge were screened for eligibility as per the published protocol [25]. Exclusion criteria included admission under the care of an older adult ward, learning disability, admission to a psychiatric intensive care unit, or forensic mental health services, or being deemed clinically unsuitable for participation by the treating clinician.

### Participant recruitment and study procedures

Potential participants were identified through routine recordings of smoking status in electronic records and referrals from ward staff and screened for eligibility. Those eligible provided informed consent and completed a baseline assessment before randomisation. Data were collected electronically via REDCap [27, 28] hosted at the University of York, paper forms returned to York Trials Unit (YTU), or telephone interviews. Follow‐ups occurred at three months and again between four and six months post‐randomisation. The latter was a pragmatic decision taken midway through the challenging recruitment phase to enable maximizing our sample size within the fixed time frame of this study. We extended the originally planned recruitment period by two months without extending the overall study duration. This meant that participants enrolled late would have to complete second follow‐up data collection by the end of month 4 (rather than month 6 as originally intended for all).

Non‐responders received at least three reminders, and participants were compensated with GBP15 shopping vouchers for each completed assessment.

### Measures

#### Feasibility of recruitment and retention

Primary feasibility outcomes included recruitment (proportion of eligible patients enrolled), retention (proportion of participants providing follow‐up data at two time points), number of Trusts enrolled and the feasibility of collecting carbon monoxide (CO) readings (proportion of randomised participants providing CO‐verified smoking status). A priori progression criteria (Table 1) to guide decision on progression to a fully powered randomised controlled trial (RCT) had been agreed with the trial steering committee based on our combined expertise in the conduct of applied mental health and tobacco dependence research [29, 30], reflecting best practice in our study context.

**TABLE 1:** SCEPTRE feasibility study progression criteria.

Criterion	Target at end of internal pilot	Final status	Green	Amber	Red
Participant Recruitment	64 participants recruited	38 (60%)	100% (64)	60–99% (38–64)	<60% (<38)
Participant Retention	> = 80% of primary outcome data available	13/38 (34.2%)	80–100% (51–64)	60–80% (38–51)	<60% (<38)
Trusts Enrolled	8	6 (80.0%)	80–100% (7–8)	60–80% (5–6)	<60% (<5)
CO Collection^a^	> = 75% of self‐reported quitters	2/3 (66.6%)	75–100%	50–75%	<50%

^a^ Sample not large enough to provide accurate judgement for this criterion.

#### Feasibility of collecting smoking‐related measures

Smoking‐related measures included CO‐verified seven‐day point prevalence abstinence (<10 ppm), self‐reported relapse, continuous abstinence, quit attempts and nicotine dependence (Heaviness of Smoking Index [31]. Additional measures were strength of urges to smoke [32], motivation to quit (Motivation to Stop Smoking Questionnaire) [33], self‐efficacy and use of e‐cigarettes and Nicotine Replacement Therapy (NRT) post‐discharge.

#### Feasibility of collecting mental and physical health measures, health‐related quality of life and resource use

Measures included the Patient Health Questionnaire depression scale (PHQ‐9) [34], the Generalised Anxiety Disorder scale (GAD‐7) [35] and the quality‐of‐life scale (EuroQol‐ 5 Dimension [EQ‐5D]) [36]. Data on health service use since discharge were also collected for health economic analysis.

#### Acceptability

Semi‐structured interviews were conducted with enrolled study participants from both groups who were contactable, interventionists, and Trust research and development (R&D) staff to explore the acceptability of study procedures and intervention delivery. Interviews followed atopic guide informed by APEASE criteria [37], were audio‐recorded with consent, and transcribed verbatim.

#### Health economics

The cost analysis followed NICE guidance from the NHS and personal social services (PSS) perspective [38], with costs reported in 2023/24 GBP. Intervention costs (development, training, app licensing) were recorded via staff time logs and trial expenses, while delivery costs were tracked using MTS–participant contact logs. Usual care costs were derived from follow‐up questionnaires. Healthcare resource use was assessed at baseline and follow‐up using a tailored questionnaire covering smoking‐related and general healthcare contacts. Health‐related quality of life was measured with the EQ‐5D‐5L [39] and data completeness was assessed.

### Randomisation and allocation concealment

Participants were randomly assigned (1:1) to the SCEPTRE intervention or usual care using a computer‐generated sequence with permuted blocks (sizes 4, 6 and 8), stratified by site. Randomisation was implemented via REDCap by Trust research staff, ensuring allocation concealment. The sequence was prepared by an independent YTU statistician. Due to the nature of the intervention, participants and site researchers were not blinded. The trial statistician conducting the analyses was also unblinded for this feasibility study.

### Intervention

The SCEPTRE intervention was a 12‐week, theory‐informed support programme that included a bespoke resource kit (the ‘MyTry kit’), tailored behavioural support with digital components (see Table 2 for summary of intervention components). It commenced prior to discharge from the in‐patient ward and was delivered by trained mental health workers known as ‘My‐Try’ Specialists (MTSs) to promote smoking cessation and prevent relapse following discharge from mental health in‐patient care. It was developed through systematic reviews, expert and stakeholder consultation, and co‐production with clinicians, tobacco control experts, and the PPI panel [18, 22, 23, 40]. Details of intervention development are reported elsewhere [23, 40]. Control group participants received usual care as per UK national guidance, which varied by site but as a minimum included signposting or support to access pharmacological and behavioural support (and sometimes e‐cigarettes) through trained smoking cessation advisors in the community post discharge. All participants were sign posted to community‐based smoking cessation services.

**TABLE 2:** Summary of individual SCEPTRE intervention components.

Intervention component	Core/additional	Aim	Frequency	Delivery
Pre‐discharge reflection, evaluation and goal setting sessions	Core	Provide personalised and tailored support to assist participants in identifying and planning their smoking‐related behaviour change goals. Discussions will also determine if participants want to use additional intervention components.	Participant‐led; may include up to two sessions lasting up to 45 minutes	MTS to deliver
Provision of a bespoke and personalised resource folder: My‐Try kit	Core	Kit including practical information and motivational content to support participants following discharge.	One kit provided pre‐discharge	MTS to provide kit
Nicotine Replacement Therapy (NRT)/e‐cigarette selection and advice	Core	To discuss preferences for NRT or e‐cigarette support as well as practical advice in relation to using the products and obtaining a longer‐term supply.	Discussed during pre‐discharge reflection and evaluation session, and MTS will continue to provide advice in behavioural support calls	MTS to deliver
Tailored behavioural support calls (via telephone or video call)	Core	Provide personalised and tailored support, assist the participant in maintaining positive change achieved during their smokefree admission and achieving their behaviour change goals, and provide feedback and encouragement on participant's progress.	Calls lasting 10–30 minutes for 12‐weeks. On the first five days post‐discharge, calls will be made daily, and weekly thereafter for the remaining eleven weeks	MTS to deliver
Smoke Free app	Additional	Provide additional support to participants, including its offer of 24/7 live smoking cessation advisor support	As required	MTS to deliver ‘onboarding’ session; smoking cessation advisors to provide advice via app
Text‐based support	Additional	Personalised text messages to provide practical and motivational information to participants based on their individual goals (Appendix 2 shows example text messages)	15–30 text messages for 8–15 days dependent on goals	MTS to deliver

#### Intervention delivery and supervision

Interventionists (MTSs) completed the National Centre for Smoking Cessation Training (NCSCT) Practitioner Training module 2, a specialty course, and bespoke training on smoking behaviour change, e‐cigarettes, and potential tobacco‐antipsychotic medication interactions, following Royal College of Psychiatrists guidance [5, 41]. Training included self‐guided online modules and an in‐person session facilitated by the research team. Monthly supervision was provided by the team, with oversight from the Trust Principal Investigator or designated individual. Fidelity was monitored through supervision, self‐assessment, interaction logs, and sampled documentation to identify training and delivery gaps.

#### Sample size

The study aimed to recruit 64 patients to allow a participation rate of 15% and completion rate of 80% to be estimated within a 95% confidence interval of ±3% and ±10% respectively [42].

### Data analyses

#### Quantitative data

Analyses were conducted in Stata v17. Baseline and follow‐up data were summarised descriptively by group and timepoint, without formal statistical comparisons [43]. Continuous data were reported as means, standard deviations, medians, and ranges; categorical data as counts and percentages. Recruitment and retention, including missing data were summarised against pilot progression criteria, with recruitment rates presented with 95% confidence intervals. The analysis plan was not pre‐registered, and the results should be considered exploratory.

#### Qualitative data

Thematic analysis [44] was conducted using NVivo v14 [45] for data management, combining deductive coding based on research questions with inductive identification of emergent themes, mapped broadly to APEASE criteria [37]. Three researchers independently coded transcripts, refined codes after initial analysis, and resolved discrepancies through discussions.

#### Health economics

The evaluation estimated intervention and usual care costs and assessed the feasibility of collecting economic data for a future trial. Using a micro‐costing approach, resource use (e.g., training, delivery, cessation aids) was costed with national unit rates (e.g. Personal Social Services Research Unit, university pay scales [46, 47]. EQ‐5D‐5L responses were converted to utility scores to calculate quality‐adjusted life years (QALYs), with descriptive statistics reported for resource use and EQ‐5D‐5L scores.

## RESULTS

### Characteristics of the trial population

Between February and July 2024, a total of 38 participants from six Trusts were randomised (17 SCEPTRE intervention and 21 usual care (Figure 1). The mean age of our sample was 37 years, and just over half were male (n = 20, 52.6%). The mean length of admission was 61.4 days (SD 52.4) with the number admitted on a voluntary or compulsory basis being similar (Table 3).

**FIGURE 1:**
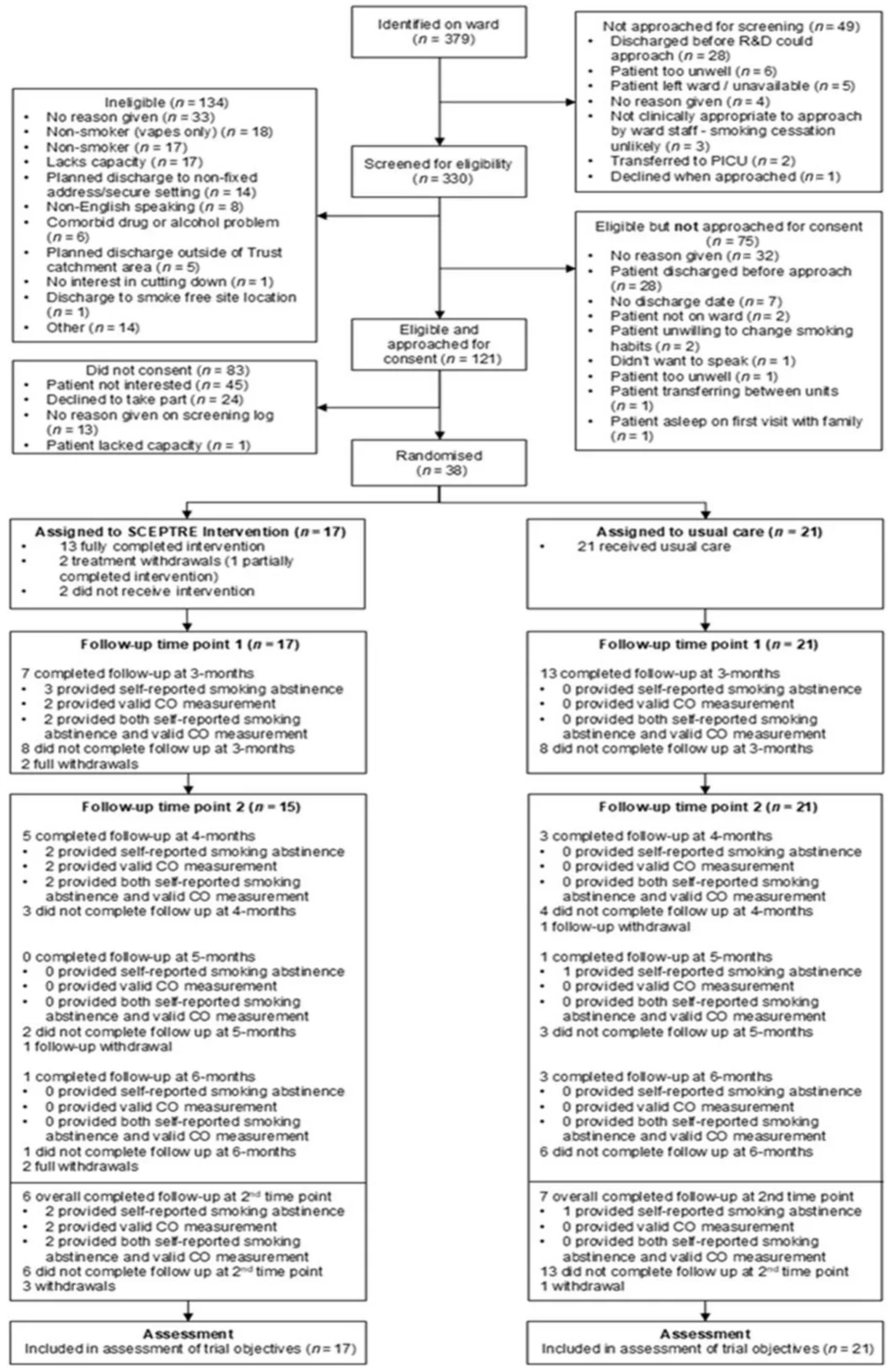
CONSORT diagram.

**TABLE 3:** Baseline demographics stratified by participant returning at least one of their follow‐up questionnaires.

	At least one questionnaire returned			No questionnaires returned		
	Intervention	Usual Care	Overall	Intervention	Usual Care	Overall
	N = 7	N = 13	N = 20	N = 10	N = 8	N = 18
Age (Years)						
N (% data available)	7 (100.0)	13 (100.0)	20 (100.0)	10 (100.0)	8 (100.0)	18 (100.0)
Mean (SD)	42.4 (11.0)	33.7 (9.2)	36.8 (10.5)	35.2 (8.0)	37.8 (12.3)	36.3 (9.9)
Median	43	32	35	35	36	36
Min, Max	21, 56	19, 54	19, 56	21, 47	24, 59	21, 59
Gender, N (%)						
Male	3 (42.9)	7 (53.8)	10 (50.0)	4 (40.0)	6 (75.0)	10 (55.6)
Female	3 (42.9)	6 (46.2)	9 (45.0)	6 (60.0)	2 (25.0)	8 (44.4)
Non‐binary	1 (14.3)	0 (0.0)	1 (5.0)	0 (0.0)	0 (0.0)	0 (0.0)
Ethnicity, N (%)						
White (any White background)	6 (85.7)	9 (69.2)	15 (75.0)	9 (90.0)	5 (62.5)	14 (77.8)
Mixed/Multiple ethnic groups	1 (14.3)	0 (0.0)	1 (5.0)	0 (0.0)	0 (0.0)	0 (0.0)
Asian/British Asian	0 (0.0)	2 (15.4)	2 (10.0)	1 (10.0)	0 (0.0)	1 (5.6)
Black/African /Caribbean/Black British	0 (0.0)	1 (7.7)	1 (5.0)	0 (0.0)	3 (37.5)	3 (16.7)
Other: Please specify	0 (0.0)	1 (7.7)	1 (5.0)	0 (0.0)	0 (0.0)	0 (0.0)
Highest educational qualification, N (%)						
GCSE/O‐level/CSE	0 (0.0)	6 (46.2)	6 (30.0)	5 (50.0)	3 (37.5)	8 (44.4)
A‐level or equivalent	4 (57.1)	3 (23.1)	7 (35.0)	2 (20.0)	3 (37.5)	5 (27.8)
Bachelor's degree or equivalent	1 (14.3)	1 (7.7)	2 (10.0)	2 (20.0)	0 (0.0)	2 (11.1)
Masters/PhD or equivalent	0 (0.0)	1 (7.7)	1 (5.0)	0 (0.0)	0 (0.0)	0 (0.0)
No formal qualifications	1 (14.3)	2 (15.4)	3 (15.0)	0 (0.0)	2 (25.0)	2 (11.1)
Other: Please specify	1 (14.3)	0 (0.0)	1 (5.0)	1 (10.0)	0 (0.0)	1 (5.6)
Employment status, N (%)						
Working: Full‐time (30 + hours per week)	1 (14.3)	2 (15.4)	3 (15.0)	3 (30.0)	0 (0.0)	3 (16.7)
Working: Part‐time (< 30 hours per week)	0 (0.0)	0 (0.0)	0 (0.0)	0 (0.0)	1 (12.5)	1 (5.6)
Working: Self‐employed	0 (0.0)	0 (0.0)	0 (0.0)	1 (10.0)	1 (12.5)	2 (11.1)
Not working: Looking for work.	0 (0.0)	0 (0.0)	0 (0.0)	0 (0.0)	2 (25.0)	2 (11.1)
Not working: Illness/disability.	6 (85.7)	9 (69.2)	15 (75.0)	6 (60.0)	2 (25.0)	8 (44.4)
Other: Please specify	0 (0.0)	2 (15.4)	2 (10.0)	0 (0.0)	2 (25.0)	2 (11.1)
Current accommodation type, N (%)						
Owned/mortgaged	1 (14.3)	0 (0.0)	1 (5.0)	0 (0.0)	0 (0.0)	0 (0.0)
Rented: Private Tenant	2 (28.6)	1 (7.7)	3 (15.0)	3 (30.0)	0 (0.0)	3 (16.7)
Rented: Social housing provider	1 (14.3)	3 (23.1)	4 (20.0)	4 (40.0)	7 (87.5)	11 (61.1)
Supported accommodation	2 (28.6)	1 (7.7)	3 (15.0)	1 (10.0)	0 (0.0)	1 (5.6)
Living with family members	1 (14.3)	6 (46.2)	7 (35.0)	0 (0.0)	1 (12.5)	1 (5.6)
Living with friends	0 (0.0)	1 (7.7)	1 (5.0)	0 (0.0)	0 (0.0)	0 (0.0)
Other: Please specify	0 (0.0)	1 (7.7)	1 (5.0)	1 (10.0)	0 (0.0)	1 (5.6)
Missing	0 (0.0)	0 (0.0)	0 (0.0)	1 (10.0)	0 (0.0)	1 (5.6)
Marital status, N (%)						
Single	5 (71.4)	9 (69.2)	14 (70.0)	9 (90.0)	6 (75.0)	15 (83.3)
Married/Civil Partner	0 (0.0)	2 (15.4)	2 (10.0)	0 (0.0)	1 (12.5)	1 (5.6)
Living with a partner/co‐habiting	0 (0.0)	1 (7.7)	1 (5.0)	1 (10.0)	0 (0.0)	1 (5.6)
Divorced/separated	2 (28.6)	1 (7.7)	3 (15.0)	0 (0.0)	1 (12.5)	1 (5.6)
Time on ward (days)						
N (% data available)	7 (100.0)	12 (92.3)	19 (95.0)	10 (100.0)	8 (100.0)	18 (100.0)
Mean (SD)	102.3 (84.6)	45.7 (15.1)	66.5 (57.6)	47.5 (29.2)	66.8 (64.0)	56.1 (47.3)
Median	96	45	48	37	35	35
Min, Max	23, 279	20, 76	20, 279	24, 115	25, 211	24, 211
Type of admission, N (%)						
Voluntary	5 (71.4)	7 (53.8)	12 (60.0)	5 (50.0)	1 (12.5)	6 (33.3)
Compulsory	2 (28.6)	6 (46.2)	8 (40.0)	5 (50.0)	7 (87.5)	12 (66.7)
Number of diagnoses, N (%)						
0	1 (14.3)	0 (0.0)	1 (5.0)	0 (0.0)	0 (0.0)	0 (0.0)
1	4 (57.1)	12 (92.3)	16 (80.0)	10 (100.0)	6 (75.0)	16 (88.9)
2	1 (14.3)	1 (7.7)	2 (10.0)	0 (0.0)	1 (12.5)	1 (5.6)
3	1 (14.3)	0 (0.0)	1 (5.0)	0 (0.0)	1 (12.5)	1 (5.6)
Diagnosis (Not mutually exclusive), N (%)						
Schizophrenia or other primary psychotic disorders	3 (42.9)	7 (53.8)	10 (50.0)	4 (40.0)	6 (75.0)	10 (55.6)
Personality disorders and related traits	3 (42.9)	2 (15.4)	5 (25.0)	5 (50.0)	2 (25.0)	7 (38.9)
Mood disorders	1 (14.3)	3 (23.1)	4 (20.0)	1 (10.0)	1 (12.5)	2 (11.1)
Mental or behavioural symptoms, signs or clinical findings	0 (0.0)	1 (7.7)	1 (5.0)	0 (0.0)	0 (0.0)	0 (0.0)
Disorders due to substance use or addictive behaviours	0 (0.0)	1 (7.7)	1 (5.0)	0 (0.0)	1 (12.5)	1 (5.6)
Neurodevelopmental disorders	1 (14.3)	0 (0.0)	1 (5.0)	0 (0.0)	0 (0.0)	0 (0.0)

### Patient recruitment and retention

Despite our best efforts, including extension of the recruitment period and attempts to enroll further mental health Trusts, we did not manage to recruit a greater sample in the given study timeframe. Seven approached Trusts were unable to participate because their usual care had recently changed to intensive support with contractual and performance management implications, making the implementation of the SCEPTRE intervention on their wards unviable at the time of study. Of 379 potential participants identified, 330 (87.1%) were screened, 196 (51.7%) were eligible, and 38 (19.4%; 95% CI: 14.1–25.6%) were randomised. Among those approached but unconsented (n = 83), 45 (54.2%) were not interested in the study, 37 (44.6%) gave no reason for declining and 1 (1.2%) lacked capacity (Figure 1). The six Trusts involved recruited an average of 1.36 (range 0.45 to 2.34) participants per site per month. Follow‐up data were obtained from 20 (52.6%) participants at 3‐months and 13 (34.2%) participants at 4 (n = 8), 5 (n = 4)‐ or 6 (n = 1)‐months.

### Progression criteria

Progression criteria for participants recruited and Trusts enrolled were rated amber (potentially viable), while participant retention was rated red (failed) (Table 1). Collection of CO readings to validate self‐reported smoking stated was rated amber, with two (66.6%) participants providing a CO reading at 3‐months post‐randomisation. However, given the exceedingly small sample (with only three participants having self‐reported as quitters at 3‐months post‐randomisation), this figure is not statistically meaningful.

### Outcome measures

#### Baseline

Table 3 provides information on the psychiatric diagnoses of participants. Over half (n = 20, 52.6%) had schizophrenia or other primary psychotic disorders, 12 (31.6%) had personality disorders, and six (15.8%) had mood disorders. Most (n = 33, 86.8%) were smoking at baseline (Table 4). One intervention participant (5.9%) and four in usual care (19%) were CO‐verified abstinent post‐admission. Nicotine dependence appeared low (mean HSI = 1.4, SD 1.8), with 17 (44.7%) reporting smoking within 60 minutes of waking and 27 (68.4%) typically smoking 10 or fewer cigarettes a day. Strength of urges to smoke was moderate (mean = 6.6, SD 2.4) and motivation to quit was high (mean = 8.9, SD 2.2). E‐cigarette use (any use, i.e., exclusive and dual) was common (n = 30, 78.9%). Mean PHQ‐9 and GAD‐7 scores indicated moderate depression (12.1 ± 7.0) and mild–moderate anxiety (9.3 ± 6.0) (Table 4).

**TABLE 4:** Baseline PROMs summarised descriptively overall and by treatment group.

	SCEPTRE intervention	Usual care	Overall
	N = 17	N = 21	N = 38
**7‐day point prevalence**			
**Self‐reported as abstinent, N (%)**			
No	16 (94.1)	17 (81.0)	33 (86.8)
Yes	1 (5.9)	4 (19.0)	5 (13.2)
**CO‐validated as abstinent, N (%)**			
No	16 (94.1)	17 (81.0)	33 (86.8)
Yes	1 (5.9)	4 (19.0)	5 (13.2)
**Continuous abstinence**			
**Length of time since last smoked, N (%)**			
0–7 days ago	16 (94.1)	17 (81.0)	33 (86.8)
2–3 weeks ago	1 (5.9)	2 (9.5)	3 (7.9)
1–2 months ago	0 (0.0)	2 (9.5)	2 (5.3)
**CO measurement**			
**CO reading collected, N (%)**			
No	1 (5.9)	1 (4.8)	2 (5.3)
Yes	16 (94.1)	20 (95.2)	36 (94.7)
**CO reading, N (%)**			
0–4 ppm	8 (47.1)	10 (47.6)	18 (47.4)
5–9 ppm	1 (5.9)	3 (14.3)	4 (10.5)
> =10 ppm	7 (41.2)	7 (33.3)	14 (36.8)
Missing	1 (5.9)	1 (4.8)	2 (5.3)
**HSI**			
**Time until first cigarette after waking up, N (%)**			
Within 5 minutes	3 (17.6)	3 (14.3)	6 (15.8)
6–30 minutes	2 (11.8)	3 (14.3)	5 (13.2)
31–60 minutes	3 (17.6)	4 (19.0)	7 (18.4)
After 60 minutes	8 (47.1)	9 (42.9)	17 (44.7)
Missing	0 (0.0)	1 (4.8)	1 (2.6)
Not applicable	1 (5.9)	1 (4.8)	2 (5.3)
**Cigarettes typically smoked each day, N (%)**			
10 or fewer	10 (58.8)	16 (76.2)	26 (68.4)
11–20 cigarettes	4 (23.5)	3 (14.3)	7 (18.4)
21–30 cigarettes	1 (5.9)	0 (0.0)	1 (2.6)
31 or more	1 (5.9)	1 (4.8)	2 (5.3)
Not applicable	1 (5.9)	1 (4.8)	2 (5.3)
**HSI Overall Score**			
N (% data available)	16 (94.1)	19 (90.5)	35 (92.1)
Mean (SD)	1.6 (2.0)	1.3 (1.6)	1.4 (1.8)
Median	1	1	1
Min, Max	0, 6	0, 6	0, 6
**Strength of urges to smoke**			
**Frequency of urges to smoke in the past 24 hrs, N (%)**			
Not at all	1 (5.9)	3 (14.3)	4 (10.5)
A little of the time	4 (23.5)	7 (33.3)	11 (28.9)
Some of the time	4 (23.5)	4 (19.0)	8 (21.1)
A lot of the time	6 (35.3)	4 (19.0)	10 (26.3)
Almost all of the time	0 (0.0)	1 (4.8)	1 (2.6)
All of the time	2 (11.8)	2 (9.5)	4 (10.5)
**Strength of urges in general, N (%)**			
Light	2 (11.8)	4 (19.0)	6 (15.8)
Moderate	9 (52.9)	10 (47.6)	19 (50.0)
Strong	2 (11.8)	3 (14.3)	5 (13.2)
Very strong	2 (11.8)	3 (14.3)	5 (13.2)
Extremely strong	2 (11.8)	1 (4.8)	3 (7.9)
**Strength of urges to smoke overall Score**			
N (% data available)	17 (100.0)	21 (100.0)	38 (100.0)
Mean (SD)	6.9 (2.2)	6.3 (2.5)	6.6 (2.4)
Median	7	6	6
Min, Max	3, 12	3, 12	3, 12
**Motivation to stop smoking**			
**Importance, N (%)**			
Not at all important	0 (0.0)	4 (19.0)	4 (10.5)
Quite important	8 (47.1)	3 (14.3)	11 (28.9)
Very important	7 (41.2)	12 (57.1)	19 (50.0)
Desperately important	2 (11.8)	2 (9.5)	4 (10.5)
**Determination, N (%)**			
Not all that determined	1 (5.9)	3 (14.3)	4 (10.5)
Quite determined	11 (64.7)	6 (28.6)	17 (44.7)
Very determined	3 (17.6)	8 (38.1)	11 (28.9)
Extremely determined	2 (11.8)	4 (19.0)	6 (15.8)
**Chance, N (%)**			
Very low	1 (5.9)	2 (9.5)	3 (7.9)
Low	1 (5.9)	2 (9.5)	3 (7.9)
Not very high	4 (23.5)	1 (4.8)	5 (13.2)
Quite high	5 (29.4)	10 (47.6)	15 (39.5)
Very high	5 (29.4)	6 (28.6)	11 (28.9)
Extremely high	1 (5.9)	0 (0.0)	1 (2.6)
**Motivation to stop smoking overall Score**			
N (% data available)	17 (100.0)	21 (100.0)	38 (100.0)
Mean (SD)	8.9 (2.1)	9.0 (2.4)	8.9 (2.2)
Median	8	9	9
Min, Max	6, 13	3, 13	3, 13
**Reasons for motivation, N (%)**			
Because my health is already suffering	3 (17.6)	4 (19.0)	7 (18.4)
Because I am worried about my future health	4 (23.5)	6 (28.6)	10 (26.3)
Because smoking costs too much	4 (23.5)	3 (14.3)	7 (18.4)
For my family's health	3 (17.6)	3 (14.3)	6 (15.8)
Other (please specify):	3 (17.6)	5 (23.8)	8 (21.1)
**E‐cigarette usage**			
**E‐cigarettes used while in hospital, N (%)**			
No	4 (23.5)	4 (19.0)	8 (21.1)
Yes	13 (76.5)	17 (81.0)	30 (78.9)
**Frequency of e‐cigarette usage, N (%)**			
Less than once a week	0 (0.0)	3 (14.3)	3 (7.9)
Less than daily, but more than once a week	0 (0.0)	1 (4.8)	1 (2.6)
Every day	13 (76.5)	13 (61.9)	26 (68.4)
Not applicable	4 (23.5)	4 (19.0)	8 (21.1)
**Length of e‐cigarette usage while in hospital, N (%)**			
Less than one week	1 (5.9)	2 (9.5)	3 (7.9)
1–2 weeks	0 (0.0)	3 (14.3)	3 (7.9)
2–3 weeks	2 (11.8)	2 (9.5)	4 (10.5)
3–4 weeks	0 (0.0)	5 (23.8)	5 (13.2)
1–2 months	3 (17.6)	2 (9.5)	5 (13.2)
2–3 months	3 (17.6)	1 (4.8)	4 (10.5)
3 months or more	4 (23.5)	2 (9.5)	6 (15.8)
Not applicable	4 (23.5)	4 (19.0)	8 (21.1)
**Type of e‐cigarette used (not mutually exclusive), N (%)**			
Single‐use ‐ Disposable	11 (64.7)	15 (71.4)	26 (68.4)
Rechargeable ‐ Pre‐filled pods or cartridges	3 (17.6)	2 (9.5)	5 (13.2)
Rechargeable ‐ Refillable (Tank)	0 (0.0)	1 (4.8)	1 (2.6)
**PHQ‐9**			
N (% data available)	17 (100.0)	21 (100.0)	38 (100.0)
Mean (SD)	12.5 (7.2)	11.8 (7.0)	12.1 (7.0)
Median	12	10	12
Min, Max	0, 26	1, 27	0, 27
**GAD‐7**			
N (% data available)	17 (100.0)	21 (100.0)	38 (100.0)
Mean (SD)	9.8 (6.8)	9.0 (5.4)	9.3 (6.0)
Median	12	9	9
Min, Max	0, 19	0, 18	0, 19

#### Three‐months follow‐up

At 3‐months follow‐up, data were collected from 20 participants (52.6%; 7 from the intervention and 13 from the usual care group). Three participants from the intervention group self‐reported seven‐day point prevalence smoking abstinence; CO validation confirmed abstinence in two cases, while the third participant did not return a CO reading and therefore was assumed not to be abstinent. In contrast, no participants in the usual care group reported point prevalence abstinence. Continuous abstinence (not smoking for over one month) was reported by three participants in the intervention group (two of these participants had their CO validation confirmed). All intervention participants reported e‐cigarette use. Mental health outcomes indicated moderate levels of depressive symptoms (PHQ‐9 M = 9.6, SD = 7.1) and mild anxiety (GAD‐7 M = 8.9, SD = 7.0), in the context of existing mental health diagnoses.

#### Four‐ to six‐months follow up

At 4–6‐month follow‐up, 13 participants (34.2%) provided outcome data (6 intervention and 7 usual care). Two participants from the intervention arm self‐reported and CO‐validated as abstinent, while one participant from the usual care group self‐reported abstinence, though this was not supported by the CO reading. Most participants (n = 10) continued using e‐cigarettes. At this timepoint, mental health symptoms were slightly elevated compared to three months, with moderate depression (PHQ‐9 M = 13.5, SD = 8.5) and moderate anxiety (GAD‐7 M = 10.3, SD = 6.4) (Table 5).

**TABLE 5:** PROMs at 3‐months and 4/5/6‐months post‐randomisation summarised descriptively by treatment group.

	3‐ months			4/5/6‐months		
	SCEPTRE intervention	Usual care	Overall	SCEPTRE intervention	Usual care	Overall
	N = 7	N = 13	N = 20	N = 6	N = 7	N = 13
**7‐day point prevalence**						
**Self‐reported as abstinent, N (%)**						
No	4 (57.1)	13 (100.0)	17 (85.0)	4 (66.7)	6 (85.7)	10 (76.9)
Yes	3 (42.9)	0 (0.0)	3 (15.0)	2 (33.3)	1 (14.3)	3 (23.1)
**CO‐validated as abstinent, N (%)**						
No	5 (71.4)	13 (100.0)	18 (90.0)	4 (66.7)	7 (100.0)	11 (84.6)
Yes	2 (28.6)	0 (0.0)	2 (10.0)	2 (33.3)	0 (0.0)	2 (15.4)
**Continuous abstinence**						
**Length of time since last smoked, N (%)**						
0–7 days ago	4 (57.1)	13 (100.0)	17 (85.0)	4 (66.7)	6 (85.7)	10 (76.9)
1–2 months ago	2 (28.6)	0 (0.0)	2 (10.0)	0 (0.0)	1 (14.3)	1 (7.7)
Not applicable	1 (14.3)	0 (0.0)	1 (5.0)	2 (33.3)	0 (0.0)	2 (15.4)
**CO measurement**						
**CO reading collected, N (%)**						
Yes	2 (28.6)	0 (0.0)	2 (10.0)	2 (33.3)	1 (14.3)	3 (23.1)
No	5 (71.4)	13 (100.0)	18 (90.0)	4 (66.7)	6 (85.7)	10 (76.9)
**CO reading, N (%)**						
0–4 ppm	2 (28.6)	0 (0.0)	2 (10.0)	2 (33.3)	0 (0.0)	2 (15.4)
> =10 ppm	0 (0.0)	0 (0.0)	0.(0.0)	0 (0.0)	1 (14.3)	1 (7.7)
Not taken	5 (71.4)	13 (100.0)	18 (90.0)	4 (66.7)	6 (85.7)	10 (76.9)
**HSI**						
**Time until first cigarette after waking up, N (%)**						
Within 5 minutes	2 (28.6)	1 (7.7)	3 (15.0)	2 (33.3)	2 (28.6)	4 (30.8)
6–30 minutes	2 (28.6)	8 (61.5)	10 (50.0)	1 (16.7)	2 (28.6)	3 (23.1)
31–60 minutes	1 (14.3)	1 (7.7)	2 (10.0)	1 (16.7)	1 (14.3)	2 (15.4)
After 60 minutes	1 (14.3)	3 (23.1)	4 (20.0)	0 (0.0)	2 (28.6)	2 (15.4)
Missing	1 (14.3)	0 (0.0)	1 (5.0)	2 (33.3)	0 (0.0)	2 (15.4)
**Cigarettes typically smoked each day, N (%)**						
10 or fewer	5 (71.4)	7 (53.8)	12 (60.0)	3 (50.0)	5 (71.4)	8 (61.5)
11–20 cigarettes	1 (14.3)	4 (30.8)	5 (25.0)	1 (16.7)	1 (14.3)	2 (15.4)
21–30 cigarettes	0 (0.0)	1 (7.7)	1 (5.0)	0 (0.0)	0 (0.0)	0.(0.0)
31 or more	0 (0.0)	1 (7.7)	1 (5.0)	0 (0.0)	1 (14.3)	1 (7.7)
Missing	1 (14.3)	0 (0.0)	1 (5.0)	2 (33.3)	0 (0.0)	2 (15.4)
**HSI Overall Score**						
N (% data available)	6 (85.7)	13 (100.0)	19 (95.0)	4 (66.7)	7 (100.0)	11 (84.6)
Mean (SD)	2.0 (1.3)	2.2 (1.6)	2.2 (1.5)	2.5 (1.0)	2.1 (2.1)	2.3 (1.7)
Median	2	2	2	3	2	3
Min, Max	0, 3	0, 6	0, 6	1, 3	0, 6	0, 6
**Strength of urges to smoke**						
**Frequency of urges to smoke in the past 24 hrs, N (%)**						
Not at all	3 (42.9)	0 (0.0)	3 (15.0)	2 (33.3)	0 (0.0)	2 (15.4)
A little of the time	1 (14.3)	5 (38.5)	6 (30.0)	0 (0.0)	3 (42.9)	3 (23.1)
Some of the time	0 (0.0)	1 (7.7)	1 (5.0)	4 (66.7)	1 (14.3)	5 (38.5)
A lot of the time	2 (28.6)	4 (30.8)	6 (30.0)	0 (0.0)	1 (14.3)	1 (7.7)
All of the time	1 (14.3)	3 (23.1)	4 (20.0)	0 (0.0)	2 (28.6)	2 (15.4)
**Strength of urges in general, N (%)**						
Zero/None	0 (0.0)	0 (0.0)	0.(0.0)	3 (50.0)	1 (14.3)	4 (30.8)
Light	4 (57.1)	3 (23.1)	7 (35.0)	1 (16.7)	0 (0.0)	1 (7.7)
Moderate	1 (14.3)	4 (30.8)	5 (25.0)	2 (33.3)	2 (28.6)	4 (30.8)
Strong	1 (14.3)	4 (30.8)	5 (25.0)	0 (0.0)	4 (57.1)	4 (30.8)
Very strong	0 (0.0)	1 (7.7)	1 (5.0)	0 (0.0)	0 (0.0)	0.(0.0)
Extremely strong	1 (14.3)	1 (7.7)	2 (10.0)	0 (0.0)	0 (0.0)	0.(0.0)
**Strength of urges to smoke overall Score**						
N (% data available)	7 (100.0)	13 (100.0)	20 (100.0)	6 (100.0)	7 (100.0)	13 (100.0)
Mean (SD)	5.7 (3.5)	7.1 (2.5)	6.6 (2.9)	4.2 (1.8)	6.9 (2.6)	5.6 (2.6)
Median	4	7	6	4	6	6
Min, Max	3, 12	4, 12	3, 12	2, 6	3, 10	2, 10
**Motivation to stop smoking**						
**Importance, N (%)**						
Not at all important	0 (0.0)	4 (30.8)	4 (20.0)	1 (16.7)	3 (42.9)	4 (30.8)
Quite important	2 (28.6)	6 (46.2)	8 (40.0)	2 (33.3)	2 (28.6)	4 (30.8)
Very important	2 (28.6)	3 (23.1)	5 (25.0)	2 (33.3)	1 (14.3)	3 (23.1)
Desperately important	3 (42.9)	0 (0.0)	3 (15.0)	1 (16.7)	1 (14.3)	2 (15.4)
**Determination, N (%)**						
Not all that determined	0 (0.0)	4 (30.8)	4 (20.0)	0 (0.0)	3 (42.9)	3 (23.1)
Quite determined	3 (42.9)	7 (53.8)	10 (50.0)	5 (83.3)	2 (28.6)	7 (53.8)
Very determined	0 (0.0)	2 (15.4)	2 (10.0)	0 (0.0)	1 (14.3)	1 (7.7)
Extremely determined	4 (57.1)	0 (0.0)	4 (20.0)	1 (16.7)	1 (14.3)	2 (15.4)
**Chance, N (%)**						
Very low	0 (0.0)	3 (23.1)	3 (15.0)	0 (0.0)	2 (28.6)	2 (15.4)
Low	0 (0.0)	3 (23.1)	3 (15.0)	0 (0.0)	1 (14.3)	1 (7.7)
Not very high	0 (0.0)	5 (38.5)	5 (25.0)	1 (16.7)	1 (14.3)	2 (15.4)
uite high	2 (28.6)	0 (0.0)	2 (10.0)	3 (50.0)	1 (14.3)	4 (30.8)
Very high	4 (57.1)	2 (15.4)	6 (30.0)	1 (16.7)	1 (14.3)	2 (15.4)
Extremely high	1 (14.3)	0 (0.0)	1 (5.0)	1 (16.7)	1 (14.3)	2 (15.4)
**Motivation to stop smoking overall Score**						
N (% data available)	7 (100.0)	13 (100.0)	20 (100.0)	6 (100.0)	7 (100.0)	13 (100.0)
Mean (SD)	11.1 (2.4)	6.4 (2.4)	8.1 (3.3)	9.2 (2.6)	7.1 (4.2)	8.1 (3.6)
Median	12	6	8	9	7	8
Min, Max	8, 14	3, 11	3, 14	6, 14	3, 14	3, 14
**Reasons for motivation, N (%)**						
Because my health is already suffering	1 (14.3)	1 (7.7)	2 (10.0)	1 (16.7)	1 (14.3)	2 (15.4)
Because I am worried about my future health	4 (57.1)	6 (46.2)	10 (50.0)	1 (16.7)	3 (42.9)	4 (30.8)
Because smoking costs too much	2 (28.6)	0 (0.0)	2 (10.0)	3 (50.0)	0 (0.0)	3 (23.1)
For my family's health	0 (0.0)	2 (15.4)	2 (10.0)	0 (0.0)	0 (0.0)	0 (0.0)
Other (please specify):	0 (0.0)	4 (30.8)	4 (20.0)	1 (16.7)	3 (42.9)	4 (30.8)
**E‐cigarette usage**						
**E‐cigarettes used while in hospital, N (%)**						
No	0 (0.0)	6 (46.2)	6 (30.0)	1 (16.7)	2 (28.6)	3 (23.1)
Yes	7 (100.0)	7 (53.8)	14 (70.0)	5 (83.3)	5 (71.4)	10 (76.9)
**Frequency of e‐cigarette usage, N (%)**						
Less than once a week	1 (14.3)	1 (7.7)	2 (10.0)	0 (0.0)	1 (14.3)	1 (7.7)
Less than daily, but more than once a week	1 (14.3)	0 (0.0)	1 (5.0)	1 (16.7)	1 (14.3)	2 (15.4)
Every day	5 (71.4)	6 (46.2)	11 (55.0)	4 (66.7)	3 (42.9)	7 (53.8)
Not applicable	0 (0.0)	6 (46.2)	6 (30.0)	1 (16.7)	2 (28.6)	3 (23.1)
**Length of e‐cigarette usage while in hospital, N (%)**						
Less than one week	1 (14.3)	0 (0.0)	1 (5.0)	1 (16.7)	1 (14.3)	2 (15.4)
1–2 weeks	0 (0.0)	0 (0.0)	0 (0.0)	0 (0.0)	0 (0.0)	0 (0.0)
2–3 weeks	1 (14.3)	1 (7.7)	2 (10.0)	0 (0.0)	0 (0.0)	0 (0.0)
3–4 weeks	0 (0.0)	0 (0.0)	0 (0.0)	0 (0.0)	0 (0.0)	0 (0.0)
1–2 months	0 (0.0)	0 (0.0)	0 (0.0)	0 (0.0)	1 (14.3)	1 (7.7)
2–3 months	5 (71.4)	6 (46.2)	11 (55.0)	0 (0.0)	0 (0.0)	0 (0.0)
‐4 months	0 (0.0)	0 (0.0)	0 (0.0)	2 (33.3)	0 (0.0)	2 (15.4)
‐6 months	0 (0.0)	0 (0.0)	0 (0.0)	2 (33.3)	3 (42.9)	5 (38.5)
ot applicable	0 (0.0)	6 (46.2)	6 (30.0)	1 (16.7)	2 (28.6)	3 (23.1)
**Type of e‐cigarette used (not mutually exclusive), N (%)**						
Single‐use ‐ Disposable	4 (57.1)	1 (7.7)	5 (25.0)	3 (50.0)	2 (28.6)	5 (38.5)
Rechargeable ‐ Pre‐filled pods or cartridges	1 (14.3)	2 (15.4)	3 (15.0)	1 (16.7)	0 (0.0)	1 (7.7)
Rechargeable ‐ Refillable (Tank)	0 (0.0)	1 (7.7)	1 (5.0)	1 (16.7)	3 (42.9)	4 (30.8)
Rechargeable ‐ Modified (Mods)	2 (28.6)	0 (0.0)	2 (10.0)	0 (0.0)	0 (0.0)	0.(0.0)
Missing	0 (0.0)	0 (0.0)	0.(0.0)	1 (16.7)	2 (28.6)	3 (23.1)
**PHQ‐9**						
N (% data available)	7 (100.0)	13 (100.0)	20 (100.0)	6 (100.0)	7 (100.0)	13 (100.0)
Mean (SD)	11.4 (7.3)	8.5 (7.1)	9.6 (7.1)	16.3 (6.1)	11.0 (9.9)	13.5 (8.5)
Median	9	7	8	19	8	17
Min, Max	2, 25	0, 27	0, 27	4, 20	1, 27	1, 27
**GAD‐7**						
N (% data available)	7 (100.0)	13 (100.0)	20 (100.0)	6 (100.0)	7 (100.0)	13 (100.0)
Mean (SD)	11.4 (7.3)	7.5 (6.7)	8.9 (7.0)	12.3 (3.9)	8.6 (7.9)	10.3 (6.4)
Median	11	6	8	14	7	9
Min, Max	1, 21	0, 20	0, 21	6, 15	1, 21	1, 21

### Adverse events and withdrawals

Three participants (one intervention, two usual care) experienced at least one non‐serious adverse event, and ten (six intervention; four usual care) had at least one serious adverse event, including one death. All involved deterioration in mental or physical health, with none deemed related to the SCEPTRE intervention. Eight participants withdrew (seven intervention, one usual care), mainly due to mental health issues or lack of interest (see Figure 1).

### Compliance with the protocol and fidelity of intervention delivery

Nine MTSs delivered 202 intervention sessions, mostly by phone (n = 149, 82.8%), including typically one (sometimes two) pre‐discharge planning sessions. Median session length was 10 minutes (IQR: 10–40; range 1–90). Session records were available from 14 (82.4%) intervention participants. All participants accepted support in the form of the My‐try kit, eleven (84.6%) accepted motivational support texts at least once, and nine (69.2%) accepted smoking app support, though only two app licences were later shown to have been actively used (Table 6).

**TABLE 6:** Information on delivery of sessions and support offered and accepted by participants.

	Session records (N = 202)
**Session details**	
**Session records**	
Total number of session records, n	202
Total number of sessions attended, n (% of session records)	180 (89.1)
**Method of session delivery, n (% of sessions attended)**	
Phone call	149 (82.8)
Face to face	15 (8.3)
Missing	16 (8.9)
**Average length of sessions, minutes**	
n	202
Mean (SD)	21.1 (19.0)
Median (IQR)	10 (6–30)
Min, Max	1, 90

	Intervention Participants (N = 17)
**Support details**	
Participants with session records, n (%)	14 (82.4)
**My‐try kit support**	
Offered at least once, n (%)	14 (82.4)
Accepted at least once, n (% of offered)	14 (100.0)
**Motivational texts support**	
Offered at least once, n (%)	13 (76.5)
Accepted at least once, n (% of offered)	11 (84.6)
**Smoking app support**	
Offered at least once, n (%)	13 (76.5)
Accepted at least once, n (% of offered)	9 (69.2)
**Reasons for not accepting support**	
**Motivational texts support, n (% of two who declined support)**	
No reason given	2 (100.0)
**Smoking app support, n (% of four who declined support)**	
No reason given	2 (50.0)
Participant stopped smoking	1 (25.0)
Participant does not have a smart phone	1 (25.0)

### Qualitative data

Six patient participants (four intervention, two usual care) were interviewed: three men and three women, mostly white (n = 5) with one unreported ethnicity. Education levels ranged from GCSEs to bachelor's degrees, and four were not working due to illness. Four MTSs and six Trust R&D representatives were also interviewed. Interviews lasted approximately 20–35 minutes each. Data is narratively presented using the most relevant aspects of the APEASE framework: Acceptability, Practicability, Effectiveness, Affordability, Safety, and Equity with verbatim quotes identified as ‘P' (patient), ‘S' (stakeholder), and ‘M'(MTS).

#### Acceptability

The SCEPTRE intervention was generally viewed as acceptable by the patients, staff and interventionists who participated in interviews. Participants valued personalised support, especially MTS contact and post‐discharge phone calls, which were described as *‘encouraging and motivating’* (P1). In contrast, the bespoke resource folder co‐produced in the early stages of the research programme (MyTry kit) and described in detail elsewhere [23] was reported to have been only rarely used, and most did not engage with the Smokefree app despite a suite of bespoke resources having been developed [40]. Advice given regarding access to and use of NRT and use of e‐cigarettes was seen as helpful for behaviour change: *‘I could start vaping then even though I'd never vaped before. That just really helped and made me just not want to go back to smoking.’* (P1). Compared with usual care, SCEPTRE was seen to offer more consistent and ‘*tailored follow‐up support’*. (S1). Stakeholders and MTSs reported positive experiences, though some said they would have preferred interactive, live training over pre‐recorded sessions: *‘With the delivery I think it was a lot of sitting in front of the computer reading a lot, even though the videos were helpful, but I think a more in person session would have been really helpful, just to ask questions on the spot and engage a bit more’*. (M2).

#### Practicability

The study was not perceived to disrupt ward operations but was viewed to pose workload challenges for some research staff and MTSs. Recruitment was experienced as difficult due to high ward acuity, fluctuating patient interest, and frequent incidents requiring staff withdrawal. Tracking discharge dates was reported to be problematic, leading to missed recruitment opportunities, which felt like ‘*a bit of a waste of our time’* (S1). Mitigation strategies used by staff included maintaining ward presence, building relationships with staff, and engaging patients in shared spaces such as the gym.

#### Effectiveness (as per APEASE acronym)

Recruitment experiences differed due to patients' varying motivation and capacity to consent. Stakeholders noted that acute mental health symptoms often limited patients' ability to prioritise research participation: ‘*when we've got things like anxiety, depression, psychosis, paranoia, it's a lot more difficult for them to attribute importance to research because they've got so much else going on’* (S1).

Most participants reported positive intervention experiences. One reported to have reduced smoking from 80 to 20 cigarettes daily, crediting NRT, vaping, and MTS support, and felt strongly about the potential positive impact for others: ‘*Please give this opportunity to other people because it's really positive … I'm really impressed with it, really pleased with it’* (P2). Another participant quit entirely and felt confident about maintaining abstinence. MTSs described cases where initially hesitant patients became motivated to address their smoking during pre‐discharge sessions.

#### Affordability

This aspect seemed of no or minor relevance for our context and was not prioritised for discussion in the interviews.

#### Safety

No urgent or harmful side effects or unintended consequences of the SCEPTRE intervention were discussed by participants. A small number of comments touched on wider patient safeguarding challenges within routine procedures, such as difficulties contacting patients and uncertainty about escalation pathways when people could not be reached: ‘*Thankfully all of mine eventually picked up and I didn't escalate it, but there were a good few weeks where I was like I can't get hold of them today’* (M1). However, these issues reflected general NHS risk management responsibilities rather than aspects of the SCEPTRE intervention itself, which did not include welfare checks or formal mental health safeguarding protocols.

#### Equity

There was some evidence of digital exclusion, for example due to limited access to the Smokefree app due to faulty devices, dyslexia, or lack of digital literacy: *‘Yeah, my phone was, it's a bit broken so I haven't been able to use it for apps or anything like that’* (P3). Many patients appeared to prefer support based on phone calls and text messages to app‐based support.

Mental health symptoms, such as paranoia, and shifting priorities post‐discharge also reduced engagement: ‘*Patients who are developing paranoid symptoms may not want to answer their phone following discharge.’* (S1).

*‘I think did we have somebody who was discharged and immediately didn't want to do the intervention. And I think he was just saying, you know, I'm moving on now with my life and I'm back at work now and … you know’*. (S2).

Financial cost rather than health often motivated quitting: *‘I think the biggest thing that came up for people wanting to come off it was the cost, rather than necessarily the health issue; it was like, I can't really afford this addiction.’* (S3). One patient described the stigma around smoking: “*I feel like a leper, everybody criticises the smoking … you know, nasty comments'* (P4) while MTS support was described as empathetic and encouraging: *‘She [MTS] was great, she was just great and with all the wobbly bits as well, that was, like, oh, I don't know, really, really wanted one, just the advice and having someone there to speak to was brilliant*.’(P1).

### Economic evaluation

Developing the SCEPTRE intervention over 18 months cost £188 104 in staff time. MTS training averaged £245 per person, rising to £625 after adjusting for substantial attrition (9 of 23 trained). Nine MTSs delivered 202 sessions to 14 participants at an average cost of £164 (SD £138) each. Additional costs included “My Try” folders (£5 each), CO monitoring (£0.50–£1.00 per use), supervision (£71 per MTS) and the Smoke Free (£39 per license and £7758 for supporting materials), though only two licenses were actively used. Of the 38 participants, 20 (52.6%) provided follow‐up data at three months and 13 (34.2%) at six months.

## DISCUSSION

### Key findings

Despite indications that the SCEPTRE intervention was perceived as acceptable by our interview respondents, we were unable to meet core progression criteria. We could not convincingly demonstrate the feasibility of implementing and evaluating the SCEPTRE intervention for mental health patients close to and immediately after discharge from acute adult mental health in‐patient wards. Smoking‐ and mental health‐related secondary outcomes were reported purely descriptively for our small sample, as the study was not powered to assess effectiveness or calculate preliminary effect sizes. Thus, most poignantly, this study highlights multiple challenges with the implementation and formal evaluation of tobacco dependence support in UK mental health in‐patient and community settings at the time of study. Some of the challenges encountered are likely to be relevant for tobacco‐focused mental health research internationally, while others may be particularly pertinent for the conduct of research in UK NHS mental health settings post Covid. We discuss our findings, with a focus on challenges encountered both at individual participant and systemic level, below.

### Feasibility

#### Recruitment and retention

We recruited 38 participants from 25 wards of six NHS mental health Trusts, demonstrating borderline feasibility of recruitment through an amber progression criterion rating. Notably, the actual recruitment rate of 19.4% was not markedly lower than predicted at study design stage (20%): of those eligible, roughly the expected proportion of participants did consent to take part. Consistent with other mental health trials [48], typical challenges arose in the context of reliably predicting and tracking time points for mental health patient discharge (which appeared to be particularly erratic in the context of extreme NHS pressures and demand on beds) and integrating study tasks into pressured clinical settings [49, 50]. However, we encountered two unexpected obstacles: 1) a substantially lower than previously evidenced smoking prevalence in patients admitted to acute adult mental health wards (we had estimated ~60% smoking prevalence on acute adult mental health wards and established that the rate was, in fact, ~32% on participating wards during our study), and 2) failure to recruit more than six mental health Trusts to our study due to recent changes in UK tobacco dependence‐related policy and practice.

In terms of over estimating prevalence rates relevant for recruitment planning, this has been shown to be a major factor in recruitment failure in clinical research, according to a systematic review of 172 RCTs from different clinical disciplines [51]. For this reason, we had based our design and recruitment plans both on research evidence relating of smoking prevalence in mental health in‐patient settings [52, 53] and on live data of smoking status recordings from four large mental health Trusts at study design stage, pre‐Covid. However, addressing smoking among people with mental health conditions has been identified as a priority to address health inequalities in the UK in recent years as set out in the UK [54], and multiple initiatives to improve support in this population were implemented between study design and implementation stage. Further research should establish if there are, in fact, widespread changes in prevalence rates that have historically been stagnating [10, 55].

Our difficulties to enrol a larger sample of mental health Trusts when recruitment was below target early in the study is related to the above. We found many Trusts ineligible or unable to take part. Based on experience with conducting the community‐based SCIMITAR trial [29], which had involved 37 community‐based NHS settings across England, we had not foreseen this. The dynamism of the tobacco dependence policy landscape in the UK meant that, as we developed the intervention and prepared the feasibility study, initiatives were taken regionally and locally to fund and implement intensive support for in‐patients in many mental health Trusts. The practical implications of this presented a major challenge, because many Trusts participating in the new schemes were also evaluating their impact and were unable to implement SCEPTRE at the same time. As such, our experience represented a common dilemma for larger study programmes with development phases that, as they evolve, may fall behind more rapid policy and practice developments, with research budgets and time frames not always flexible enough to adapt optimally.

While recruitment proved highly challenging but still received a potentially viable ‘amber’ rating, retention of participants in SCEPTRE was a clear failure: only 34.2% of all participants completed the second follow‐up, and fewer in the intervention arm. Our efforts, which included co‐developed study procedures and materials (including a bespoke co‐produced bias‐free language resource to support recruitment to SCEPTRE), weekly trouble shooting sessions with all settings, and multiple amendments to the protocol to accommodate local Trust idiosyncrasies for smoother recruitment, did not result in satisfactory retention of enrolled participants over time. Attrition was already very high (~50%) at the first follow‐up point. This experience is in stark contrast to other trials in the field, both from the UK [30] and other countries [56, 57], but in line with experiences from a recent UK‐based e‐cigarette‐focused cessation and harm‐reduction trial in community mental health patients that had to be discontinued due to low recruitment (Kale et al., manuscript under review). It is difficult to draw on insights from our qualitative interviews due to the small sample and selection bias of those who remained contactable throughout the study. However, a comment by a member of staff at interview about the difficulties of retaining patients who are still ‐ or again ‐ very unwell after discharge may point to one core explanatory factor. In the UK, in‐patient treatment now occurs as a last resort when specialist support in the community is no longer viable, and only the most severely and acutely unwell mental health patients are admitted to it [58]. Bed pressure is high, and discharge occurs as soon as clinically justifiable, with high readmission rates [59]. Many patients deteriorate after discharge. This poses extreme challenges for the development of successful retention strategies for mental health patients around discharge and, in fact, raises question about the timing of interventions for research and clinical practice. In our case, where the purpose of the intervention was inextricably linked to the point of discharge (and the first three days thereafter), it is difficult to draw conclusions.

As high attrition is common in smoking cessation trials [60], and may be worse among individuals with mental health disorders, updating and building on quantitative and qualitative evidence synthesis on effective retention strategies [61, 62] and co‐producing tailoring strategies to pressured NHS contexts could strengthen future studies.

#### Secondary outcome measures

Data collection in those who responded to contact attempts proved generally feasible, with most participants providing demographic, mental health, and economic information. High dropout rates limited the collection of smoking‐related outcomes over time. Qualitative and quantitative data indicated minimal contamination, as no usual care participants reported quitting or reducing smoking.

Abstinence rates in the intervention group at 3 and 4–6 months suggest some short‐term quitting among a small subset of participants, supported by CO validation. However, very few sustained abstinence, and high attrition underscored the difficulty of cessation among individuals with severe mental illness. The frequent use of e‐cigarettes reflects current harm reduction trends, highlighting the need to explore the role of vaping for cessation and harm reduction in this study population further.

Mental health outcomes showed ongoing moderate depressive and mild to moderate anxiety symptoms, likely affecting engagement and quit success in addition to symptoms experienced in the context of participants' primary mental health diagnoses. Minimal change over time suggests either limited intervention impact on psychiatric symptoms or delayed mental health improvements compared to behavioural change.

Economic data revealed substantial development and delivery costs, with high MTS turnover increasing adjusted training costs. The high turnover reflected intense NHS staff pressures with frequent changes to staffing and internal reorganisation at the time of our study. While the importance of careful staff selection, strong supervision, and workload support cannot be underestimated, systemic pressures outside of the influence of our research team did play a central part in our experience and continue to date.

Despite substantial investments in resources such as the Smoke Free app, which incurred costs for licenses, training and support, including a co‐produced tailored on‐boarding session [23], its usage was limited, suggesting the need to reconsider its role in a fully‐powered trial as well as in practice. Participant follow‐up rates were low, which may have impacted the accuracy of resource use reporting. However, the improvements in quality of life observed in both groups support the feasibility of incorporating health economics data in a future full‐scale trial, with further refinement needed to optimise cost‐effectiveness evaluation in a full‐scale trial.

### Acceptability

Qualitative interviews indicated that participants who completed follow‐up and remained contactable viewed the intervention, including MTS support, NRT, and e‐cigarettes, positively. The views captured will predominantly reflect those who remained engaged, excluding voices of participants who disengaged earlier and whose experiences might have revealed important barriers to sustained participation. High dropout rates raise questions about overall acceptability. Aspects relating to potential digital exclusion, low literacy levels, or technological barriers (given the low usage of certain elements such as the My‐Try kit and the Smokefree app) will be important to explore further. A participant comment about preferring in‐person support to ‘spending a lot of time in front of screens' should be further explored. The possibility of digital exclusion is particularly important considering the increasingly rapid digitalisation of health care [63], the adoption of apps, including the Smokefree app, in multiple NHS contexts, and evidence of the digital exclusion in mental health populations [64]. Because interview data likely reflect only those who stayed engaged, findings should be interpreted cautiously. Future studies should assess acceptability among disengaged participants and adapt digital components to better meet diverse needs. Strengthening retention strategies and focusing on high‐impact, personalised support that enables building relationships will be essential.

MTSs highlighted the need for enhanced training, preferably via live video sessions, and continued supervision to boost confidence and fidelity. Safety and risk management remained critical concerns, especially given the recognised relapse and suicide risk post‐discharge. While no harmful intervention effects were noted, some staff expressed uncertainty about how risk‐management protocols for the study could be aligned with general risk management procedures (e.g., when patients are not contactable). Recognising these complexities and the heightened post‐discharge risk [59, 65], additional supervision and training would be valuable for future trials to ensure consistent and safe intervention delivery.

### Safety – adverse events

Adverse events and withdrawals from enrolment were notable and reflected expected deterioration in mental or physical health within this high‐risk population. Events occurred in both trial arms, with no evidence of a causal link to the SCEPTRE intervention. Such rates align with known vulnerabilities during the post‐discharge period, a time marked by elevated risk of relapse and suicide [66]. This has been corroborated in national surveys, which highlight increased rates of immediate post‐discharge suicide and self‐harm, particularly among those with complex mental health needs and those who self‐initiate discharge [67].​ Most adverse events involved mental health deterioration, underscoring the difficulty of delivering behavioural interventions in this context and the need for strong post‐discharge support. Withdrawals were mainly due to mental health issues or loss of interest, highlighting engagement challenges among acutely unwell participants. Future trials should anticipate these risks and embed proactive wellbeing measures, including rapid follow‐up and robust safety planning.

### Implications and further research

Our findings indicate that a fully powered trial is not feasible at this point. Implications for future research include: 1) the establishment of reliable smoking prevalence rates in mental health in‐patient populations to enable effective recruitment planning; 2) research to explore strategies to improve retention of peri‐discharge mental health patients in research and clinical interventions, including co‐production of strategies that take into account both intense systemic pressures and individual characteristics of our patient group and (diagnostic) sub groups within it; 3) a review of research and intervention delivery processes that minimise participant burden in this population; and 4) further exploration of matters relating to potential digital exclusion in our study population, especially in the context of widespread implementation in healthcare systems across the world.

### Strengths and limitations

This study's main strength lies in its focus on an underserved, high‐risk population and its contribution to evidence on smoking cessation in mental health settings. Involving multiple NHS Trusts across diverse regions enhances generalisability, while the mixed‐methods design offers rich contextual insights. Limitations include the small sample size, high dropout rates, and variability in uptake of individual intervention delivery. Economic data collection was feasible, though further work is needed to refine cost‐effective components for future trials.

## CONCLUSION

The SCEPTRE feasibility study offered valuable insights into delivering smoking cessation support for individuals discharged from smoke‐free mental health settings. However, key feasibility criteria were not met, indicating that further research and refinement are required in this space. Smaller‐scale studies should address recruitment, retention, and intervention optimisation to ensure trial viability. As digital health use expands across the NHS, it is crucial to prevent digital exclusion and design interventions that promote equitable access for people with complex mental health needs.

## SCEPTRE COLLABORATORS

Lesley Colley (1), Samantha Brady (2), John Britton (3), Simon Gilbody (4, 5), Steve Parrott (2), Peter Coventry (6), Gregor Russell (7), Paul Galdas (4)

1. Research and Development, Tees, Esk and Wear Valleys NHS Foundation Trust, Darlington, UK
2. York Trials Unit, Department of Health Sciences, University of York, UK
3. University of Nottingham, UK
4. Mental Health and Addiction Research Group, Department of Health Sciences, University of York, UK
5. Hull York Medical School, University of York, York, UK
6. School of Nursing and Public Health, Manchester Metropolitan University, Manchester, UK
7. Research and Knowledge Service, Bradford District Care NHS Foundation Trust, Bradford, UK

## AUTHOR CONTRIBUTIONS

**Petal Petersen Williams:** Data curation; investigation; formal analysis; project administration; writing—original draft; validation. **Lisa Huddlestone:** Data curation; investigation; validation; formal analysis; project administration; writing—review and editing. **Emily Shoesmith:** Validation; formal analysis; data curation; writing—review and editing; investigation. **Jennifer Sweetman:** Investigation; validation; formal analysis; data curation; writing—review and editing; project administration. **Heidi Stevens:** Investigation; validation; formal analysis; data curation; writing—review and editing; project administration. **Alex Mitchell:** Investigation; methodology; validation; formal analysis; writing—review and editing. **Fraser Wiggins:** Investigation; methodology; validation; formal analysis; writing—review and editing. **Lesley Sinclair:** Investigation; methodology; supervision; writing—review and editing. **Victoria Exley:** Investigation; project administration; writing—review and editing. **Catherine Hewitt:** Methodology; supervision; writing—review and editing. **Judith Watson:** Methodology; supervision; writing—review and editing. **Qi Wu:** Investigation; methodology; validation; formal analysis; writing—review and editing. **Jodi Pervin:** Investigation; project administration; writing—review and editing; data curation. **Moira Leahy:** Investigation; methodology; visualization; writing—review and editing. **Claire Paul:** Investigation; methodology; visualization; writing—review and editing. **Lion Shahab:** Methodology; writing—review and editing; validation. **Michelle Horspool:** Conceptualization; methodology; funding acquisition; writing—review and editing; project administration; supervision. **Tim Coleman:** Methodology; validation; writing—review and editing. **Simon Hough:** Conceptualization; methodology; resources; supervision; investigation; writing—review and editing. **Elena Ratschen:** Conceptualization; investigation; funding acquisition; writing—review and editing; visualization; methodology; validation; supervision; resources; data curation.

## DECLARATION OF INTERESTS

LSh has received honoraria for talks, an unrestricted research grant and travel expenses to attend meetings and workshops from Pfizer and an honorarium to sit on advisory panel from Johnson & Johnson, both pharmaceutical companies that make smoking cessation products. He has acted as paid reviewer for grant awarding bodies and as a paid consultant for health care companies. Other research has been funded by the Department of Health, UKRI, a community‐interested company (National Centre for Smoking Cessation) and charitable sources (Cancer Research UK, Yorkshire Cancer Research). He has never received personal fees or research funding of any kind from alcohol, electronic cigarette or tobacco companies. All other authors declare no conflicts of interest.

## ETHICS APPROVAL

Ethics approval was obtained from the North West ‐ Greater Manchester West Research Ethics Committee (REC) (23/NW/0312).

## DATA SHARING

Data supporting the findings of this study are available from the Chief Investigator on reasonable request.

## TRIAL REGISTRATION WITH ISRCTN

ISRCTN77855199 (https://www.isrctn.com/ISRCTN77855199) 17/11/2023.

## Supporting information


**Data S1.** Supporting Information.

## Data Availability

The data that support the findings of this study are available from the corresponding author upon reasonable request.
